# *Neurotrophic Receptor Tyrosine Kinase 2* (*NTRK2*) Alterations in Low-Grade Gliomas: Report of a Novel Gene Fusion Partner in a Pilocytic Astrocytoma and Review of the Literature

**DOI:** 10.1155/2020/5903863

**Published:** 2020-01-30

**Authors:** Siobhan S. Pattwell, Eric Q. Konnick, Yajuan J. Liu, Rebecca A. Yoda, Laligam N. Sekhar, Patrick J. Cimino

**Affiliations:** ^1^Division of Human Biology, Fred Hutchinson Cancer Research Center, Seattle, WA, USA; ^2^Department of Laboratory Medicine, University of Washington, Seattle, WA, USA; ^3^Department of Pathology, Division of Neuropathology, University of Washington, Seattle, WA, USA; ^4^Department of Neurological Surgery, University of Washington, Seattle, WA, USA

## Abstract

Pilocytic astrocytoma is a low-grade glial neoplasm of the central nervous system (CNS) that tends to occur in the pediatric population and less commonly presents in adults. Hereditary pilocytic astrocytoma is often associated with germline genetic alterations in the tumor suppressor *NF1*, the gene responsible for the syndrome neurofibromatosis type 1. Sporadic pilocytic astrocytoma frequently harbors somatic alterations in *BRAF*, with rare pilocytic astrocytomas containing alterations in *FGFR1* and *NTRK2*. *NTRK2* encodes for the protein tropomyosin receptor kinase B (TrkB), which is a neurotrophin receptor with high affinity for Brain-Derived Neurotrophic Factor (BDNF), and plays a role in several physiological functions of neurons, including cell survival and differentiation. In this report, we describe a novel *PML-NTRK2* gene fusion occurring in an adult sporadic pilocytic astrocytoma and review the biology and implications of specific *NTRK2* mutations occurring in CNS neoplasms.

## 1. Introduction

Pilocytic astrocytoma, World Health Organization (WHO) grade I, is the most common low-grade glial neoplasm found in the pediatric population and is infrequent in adults. Pilocytic astrocytoma can occur in the setting of hereditary disorders, such as neurofibromatosis type 1 (NF1), or sporadically in the absence of any tumor predisposition syndrome. The most common (observed in 15-20% overall) intracranial neoplasm in pediatric NF1 patients is the optic pathway glioma, which usually is classified as pilocytic astrocytoma and typically presents in children younger than 7 years of age [1–3]. NF1 results from germline mutations in the *NF1* tumor suppressor gene, and pilocytic astrocytoma associated with NF1 additionally contain biallelic inactivation of NF1 and loss of expression of the *NF1* protein product (neurofibromin) [4]. Sporadically occurring pilocytic astrocytoma frequently contains somatic alterations in the *BRAF* gene, which encodes for a serine/threonine kinase also involved in the RAS/MAPK/ERK signaling pathway [5]. Unlike the loss-of-function mutations found in *NF1*, *BRAF* alterations predominantly occur as an oncogenic gene fusion product, *KIAA1549-BRAF* [5]. Outside of the frequent alterations occurring in *NF1* and *BRAF* genes, very rare genetic alterations in *FGFR1* and *NTRK2* have been reported [6]. To our knowledge, *NTRK2* (the gene that encodes for the neurotrophin receptor TrkB) alterations have been described in only eight cases of low-grade circumscribed gliomas, with half (*n* = 4) being associated with pilocytic astrocytomas, which are resultant from gene fusions with partners including *QKI*, *NACC2*, and *KANK1* (Table 1) [6, 7]. Additionally, a single case of a low-grade diffuse glioma has been reported with an *NTRK2* gene fusion (Table 1) [8]. *NTRK2* fusion partners in nonpilocytic astrocytoma low-grade gliomas include *QKI*, *SLMAP*, *TLE4*, *NAV1*, and *BCR* [8–11]. Here, we describe a patient where a novel *PML-NTRK2* gene fusion was identified in an adult sporadic pilocytic astrocytoma. The *PML* gene itself is a transcription factor that is associated with promyelocytic leukemia, and such alterations have not been reported in pilocytic astrocytoma. In addition to expanding the landscape of *NTRK2* mutations occurring in the setting of pilocytic astrocytoma, we review the biological and therapeutic implications of altered TrkB signaling in low-grade glial neoplasms.

## 2. Case Presentation

**Figure 1:**
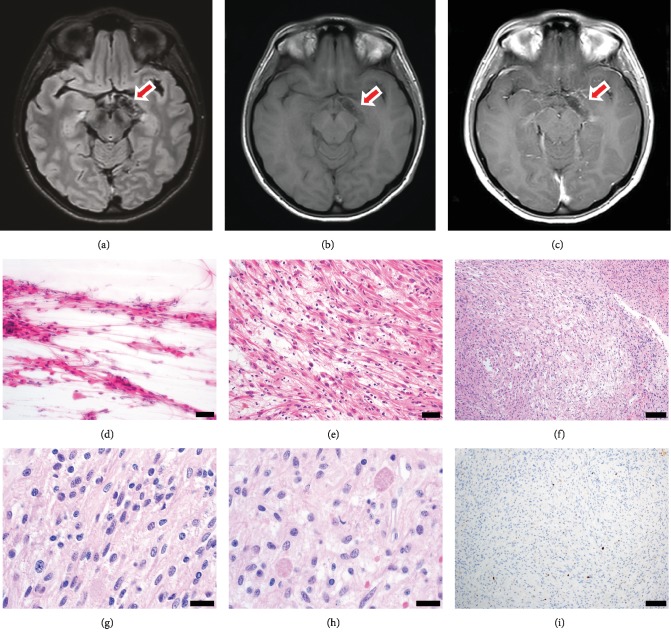
Pilocytic astrocytoma radiographic and histopathologic features. Brain magnetic resonance imaging demonstrates T2/FLAIR curvilinear hyperintensity (a) and T1 hypointensity (b) without contrast enhancement (c). Hematoxylin and eosin-stained intraoperative cytological smear preparation (d) and frozen section (e) demonstrating an astrocytoma with thin elongated piloid (“hair-like”) cellular processes (scale bar = 50 microns). (f) Permanent sections showed a piloid astrocytoma with compact areas and adjacent slightly looser areas (scale bar = 100 microns). (g) The astrocytic nuclei have mild pleomorphism and are elongated, slightly irregular, and hyperchromatic (scale bar = 20 microns). (h) Abundant eosinophilic granular bodies are found throughout the astrocytoma (scale bar = 20 microns). (i) The Ki-67 proliferative index is low (<1%) overall (scale bar = 100 microns).

The patient is a 33-year-old woman with past medical history of hypothyroidism and gastroesophageal reflux disease, who has had chronic headaches refractory to medical management. A maxillofacial computed tomography (CT) evaluation for possible sinusitis noted an abnormal curvilinear “calcification” involving the left suprasellar cistern and mesial temporal region. Follow-up brain magnetic resonance imaging (MRI) demonstrated an abnormal T1-weighted hypointense, T2-weighted/FLAIR hyperintense, nonenhancing lesion in the area of the left suprasellar cistern and mesial temporal lobe, with mass effect over the optic tract (Figures 1(a)–1(c)). The radiographic impression included a wide differential including vascular malformation or neoplasm. She subsequently underwent left frontotemporal craniotomy and orbital osteotomy for excision of the lesion.

Hematoxylin and eosin- (H&E-) stained slides of intraoperative cytologic smear preparation and frozen section tissue demonstrated a glial neoplasm with piloid features (Figures 1(d) and 1(e)). Permanent H&E-stained sections showed multiple fragments of a solid-growing, biphasic, glial neoplasm characterized by compact piloid areas comprised of bipolar cells with bland, ovoid to elongate nuclei adjacent to more loosely arranged areas comprised of cells with angular and hyperchromatic nuclei in a fibrillary background (Figures 1(f)–1(g)). Eosinophilic granular bodies were frequently noted throughout the neoplasm, with occasional scattered Rosenthal fibers (Figure 1(h)). There were occasional hyalinized vessels as well as vessels cuffed by small lymphocytes. There was no necrosis. Abundant fresh hemorrhage and clusters of hemosiderin-laden macrophages were present. Mitotic activity was not identified, and the Ki-67 proliferative index was less than 1% overall (Figure 1(i)). Neurofilament highlighted a predominantly solid growth pattern (not shown). The overall histopathological features are diagnostic for pilocytic astrocytoma, WHO grade I. Ancillary fluorescence in situ hybridization (FISH) was negative for *BRAF* duplication or rearrangement.

**Figure 2:**
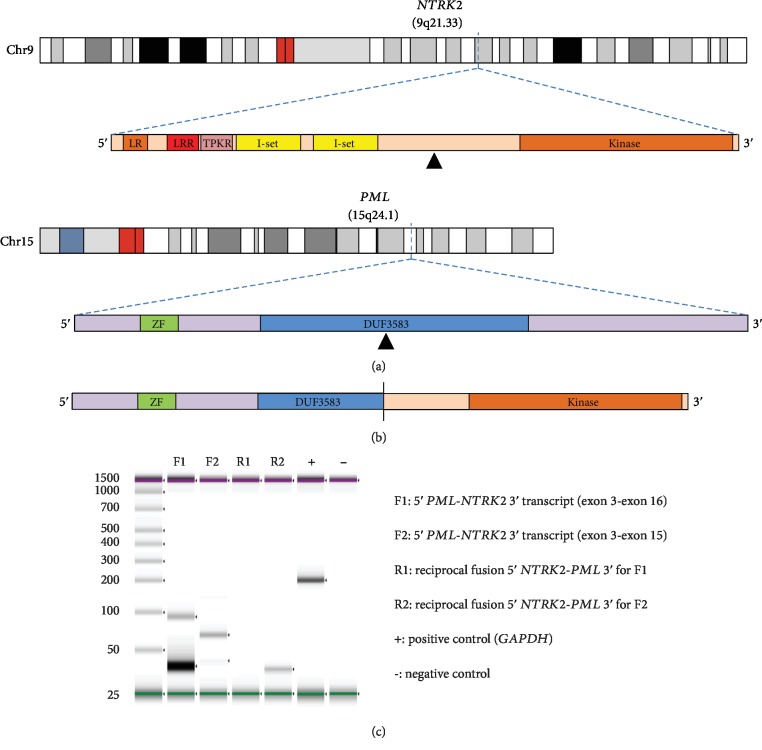
Pilocytic astrocytoma *PML*-*NTRK2* gene fusion. (a) Illustrations of the breakpoints in *NTRK2* on chromosome 9 and *PML* on chromosome 15, and associated transcripts, as determined by DNA next-generation sequencing (NGS). (b) Illustration of in-frame *PML*-*NTRK2* gene fusion product confirmed with FusionPlex RNA NGS. (c) RT-PCR analysis validating *PML-NTRK2* in-frame fusions F1 and F2. F1 = fusion transcript 1 with exon 3 of *PML* (5′ end partner) and exon 16 of NTRK2 (3′ end partner); F2 = fusion transcript 2 with exon 3 of *PML* (5′ end partner) and exon 15 of NTRK2 (3′ end partner). There are no detectable reciprocal 5′-*NTRK2-PML*-3′ fusion products.

The clinically validated UW-OncoPlex [12] next-generation sequencing (NGS) assay was used to examine 262 cancer-associated genes in the neoplastic tissue. Average target coverage for the tested sample was 577-fold, with no single-nucleotide variants (SNVs), insertion-deletion (indel), or structural mutations identified in other pilocytic astrocytoma-related genes including *BRAF*, *NF1*, and *FGFR1*. Also of note, there were no alterations detected for *IDH1*, *IDH2*, *TP53*, or *ATRX*. A novel translocation between *PML* and *NTRK2* is identified by multiple bioinformatics pipeline programs CREST [13] and BreakDancer [14], with approximate genomic breakpoints of HG19 chr9:g.87467299 with chr15:g.7431663 and chr15:g.74316451 with chr9:g.87467483. BLAT (BLAST-like alignment tool) analysis of the consensus sequence mapped uniquely to *PML* and *NTRK2* was employed, and split-read sequences were readily identified in the sequencing BAM file using the Integrative Genomics Viewer [15, 16] (IGV, Broad Institute, Cambridge, MA, USA) (Figure 2). The consensus-read data indicates that this rearrangement occurs within *NTRK2* intron 14, which is upstream of the kinase domain, and *PML* intron 3. While at the DNA level, the functional consequences of this rearrangement are not known, the newly juxtaposed exons are predicted to be in-frame for both the *NTRK2-PML* and *PML-NTRK2* rearrangements, if the splicing within the fusion gene products are not disrupted, suggesting that the genomic rearrangement may be a balanced translocation. Other glial neoplasms that have been identified to harbor *NTRK1*, *NTRK2*, or *NTRK3* fusions have been described with similarly structured rearrangements [17]. The clinically validated FusionPlex (ArcherDx, Inc., Boulder, CO, USA) NGS analysis using a custom 114-gene solid tumor panel with RNA extracted from the tumor detected a fusion between genes *PML* (5′ partner) and *NTRK2* (3′ partner). Two isoforms of fusion transcripts in-frame were detected, and both had exon 3 of *PML* (NM_002675.3) at the 5′ end with one isoform having exon 16 of *NTRK2* (NM_006180.3) at the 3′ end and the other isoform having exon 15 of *NTRK2* (NM_006180.3) at the 3′ end. For confirmation of the novel fusions discovered by NGS analysis, RT-PCR analysis was also performed for both 5′*PML-NTRK2* 3′ fusion products and potential reciprocal 5′*NTRK2-PML* 3′ fusion products. By RT-PCR, the two in-frame fusion isoforms of 5′*PML-NTRK2* 3′ were detected, while there were no detectable reciprocal 5′*NTRK2-PML* 3′ fusion products (Figure 2(c)). So while DNA analysis indicates that the *PML* and *NTRK2* rearrangement may result in balanced fusion products, at the RNA level, only 5′*NTRK2-PML* 3′ fusion products were detectable.

The patient has had 26 months of clinical follow-up and is currently without symptoms or radiographic evidence of disease.

## 3. Discussion

Pilocytic astrocytomas are typically associated with alterations in either *NF1* or *BRAF*, and less commonly in *FGFR1*. Rare somatic *NTRK2* gene fusions have been previously described in pediatric low-grade neuroepithelial tumors, including pilocytic astrocytoma and ganglioglioma (summarized in Table 1), but the incidence in adults has not been described. In the current case, there was a lack of genetic alterations in *NF1* and *BRAF*, but rather this pilocytic astrocytoma harbored a novel *NTRK2* fusion with interesting implications for biology and therapeutics. TRK inhibitors have been proposed as therapeutic targets in neoplasms with activating *NTRK* fusions [18, 19] with promising results in early-stage clinical evaluations [20, 21]. To our knowledge, this is the first report of a *NTRK2* gene fusion product in a pilocytic astrocytoma that has *PML-NTRK2*.

Located on chromosome 9 and spanning more than 350,000 base pairs (bp), *NTRK2* encodes for numerous transcripts with precise developmental and tissue-specific precision [22–24]. The initial 5′ exons exhibit complex splicing patterns with varied transcriptional start sites while also forming an internal ribosomal entry site (IRES) to the translational start site while subsequent exons encode for the extracellular domain containing several IG-like domains and a neurotrophin binding site. A single exon encodes for the transmembrane domain, which is shared between multiple transcripts, followed by several exons encoding intracellular domains with the most 3′ exons encoding for the kinase domain. To date, known *NTRK2* fusions observed in low-grade neuroepithelial tumors (summarized in Table 1), as well as diffuse intrinsic pontine glioma and other higher-grade gliomas [17], have resulted in fusions containing exons encoding for the TrkB kinase domain, postulating constitutive kinase activation and subsequent downstream signaling as the oncogenic driver. Additional gliomas with unspecified histological classification or grading have been reported as well [25].

**Figure 3:**
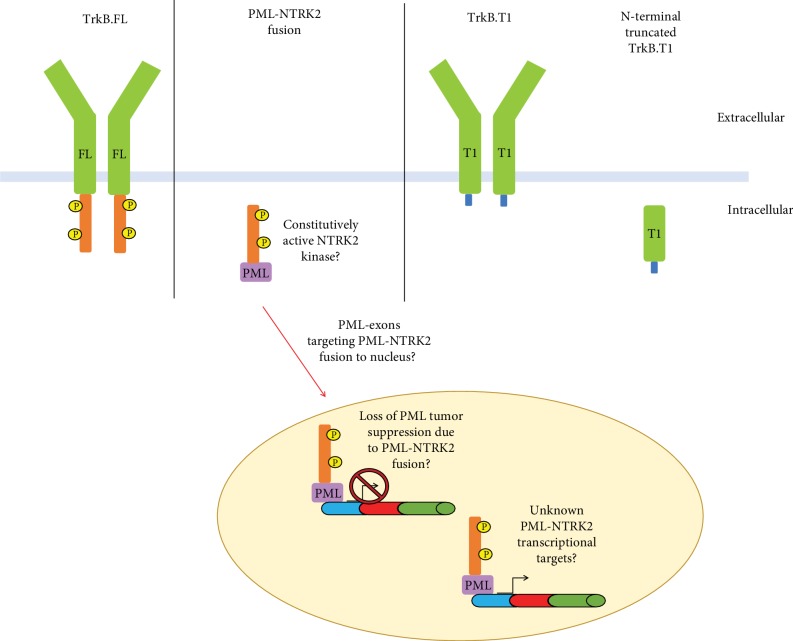
Predicted TrkB receptor outcomes encoded by *NTRK2* fusions in pediatric low-grade glioma. FL = full-length variant; KD = kinase domain; T1 = known alternative splice variant; P=phosphorylation site.

Similar to previously published TRK fusions, the *PML-NTRK2* fusion described here results in an *NTRK2* fusion harboring the TrkB kinase domain, which is of interest biologically for downstream signaling (Figure 3). Additionally, this fusion contains the first three exons of PML encoding the TRIpartite motif responsible for binding DNA. PML fusions have been well characterized in other cancer types, particularly the PML-RAR alpha oncofusion in acute promyelocytic leukemia, and are typically associated with loss-of-function mutations in the PML protein, leading to subsequent loss of tumor suppressor roles.

Given *NTRK2*'s uniquely complex splice patterns, it is also of interest to note that the fusions occur downstream of exons that may still allow for multiple kinase-deficient *NTRK2* transcripts. In addition to the full-length receptor tyrosine kinase, TrkB, several alternatively spliced *NTRK2*variants have been discovered, including TrkB.T1, TrkB-Shc, and N-terminal-truncated-TrkB.T1 (Figure 3) [22]. Once thought to be a dominant-negative/decoy receptor due to its lack of a kinase domain, one truncated splice variant, TrkB.T1, shares the same extracellular and transmembrane domains and first 12 intracellular amino acids as other variants [26]. Lacking a kinase domain, TrkB.T1 has a specific C-terminal sequence of 11 amino acids that is 100% conserved across species from rodents to humans [22, 24, 26, 27], and structure-function analysis confirms that this sequence is required for TrkB.T1 activity [28], supporting an evolutionary role beyond a dominant negative. This alternatively spliced receptor variant has been previously shown to alter Ca^2+^ signaling in glial cells in response to BDNF signaling [29] and mediate signal transduction *in vitro* [30]. Additionally, TrkB.T1 has been shown to regulate neuronal [31] and astrocytic morphology via rho GTPases [32] and outgrowth of filopodia in neuroblastoma cells [33] and contribute to glioma cell proliferation *in vitro* [32, 34]. The *NTRK2* fusion point observed here (chr9:g.87467299) is within an intron just downstream of the TrkB.T1 alternate exon (which terminates at chr9:87430621) which contains multiple poly-A sites, regulatory elements, and a stop codon, highlighting the potential that while TrkB kinase activation is not possible, an intact form of truncated TrkB.T1 (NM_001007097), among other *NTRK2* transcripts, may also be functionally expressed in conjunction with this fusion.

Basic scientific and clinical investigation surrounding Trks' precise role in cancer has long been hindered due to nonspecific of pan-Trk antibodies and kinase inhibitors alike. Current clinical trials are also plagued by nonspecificity when it comes to exploring Trks. For example, the small-molecule LOXO-01 has been designed to block the ATP binding site of Trks (including TrkA, TrkB, and TrkC) and has shown promising outcomes in clinical trials for other cancer types, but as this drug lacks specificity, its mechanism of action remains unclear [19]. The combined use of pan-Trk detection reagents for research and pan-Trk inhibitors in clinical trials may offer marginal insight into their biology. Further investigation is needed in order to hone in on precise signaling relevant to tumor biology in an effort to avoid off-target effects from multi-Trk inhibition, especially those involving the pediatric nervous system, as neurotoxic damage could have lifelong consequences. Due to the nonspecificity of the current pan-Trk kinase inhibitors, additional research confirming the kinase activity of the observed *NTRK1*, *NTRK2*, or *NTRK3* fusions is warranted. Future exploration into the role of alternative splicing of Trk receptors, and *NTRK2* specifically, will be paramount to understanding tumor biology and subsequently designing appropriate therapeutics and diagnostic approaches.

## Figures and Tables

**Table 1:** Summary of reported *NTRK2* gene fusion alterations in low-grade neuroepithelial tumors. PA = pilocytic astrocytoma; GG = ganglioglioma; DNT = dysembryoplastic neuroepithelial tumor; LGG-NOS = low-grade glioma not otherwise specified.

5′ fusion gene	3′ fusion gene	Number of cases	Histology	Reference
*QKI*	*NTRK2*	2	PA	Jones DT, 2013
*NACC2*	*NTRK2*	1	PA	Jones DT, 2013
*KANK1*	*NTRK2*	1	PA	Lopez GY, 2019
*PML*	*NTRK2*	1	PA	Current patient
*SLMAP*	*NTRK2*	1	GG	Qaddoumi I, 2016
*TLE4*	*NTRK2*	1	GG	Prabhkaran N, 2018
*NAV1*	*NTRK2*	1	DNT	Qaddoumi I, 2016
*QKI*	*NTRK2*	1	LGG-NOS	Solomon JP, 2019
*BCR*	*NTRK2*	1	Diffuse LGG-NOS	Jones KA, 2019
